# Development of three-dimensional preoperative planning system for the osteosynthesis of distal humerus fractures

**DOI:** 10.1186/s12938-020-00801-3

**Published:** 2020-07-13

**Authors:** Yuichi Yoshii, Shin Teramura, Kazuki Oyama, Takeshi Ogawa, Yuki Hara, Tomoo Ishii

**Affiliations:** 1 Department of Orthopaedic Surgery, Tokyo Medical University Ibaraki Medical Center, 3-20-1 Chuo, Ami, Inashiki, Ibaraki 300-0395 Japan; 2 Department of Orthopaedic Surgery, University of Tsukuba Hospital, Tsukuba, Ibaraki 305-8576 Japan

**Keywords:** Three-dimensional, Preoperative plan, Distal humerus fracture, Computed tomography, Osteosynthesis

## Abstract

**Background:**

To reproduce anatomical reduction and appropriate implant placement/choices during osteosynthesis for elbow fractures, we developed a 3D preoperative planning system. To assess the utility of 3D digital preoperative planning for the osteosynthesis of distal humerus fractures, we evaluated the reproducibility of implant reduction shapes and placements in patients with distal humerus fractures.

**Methods:**

Twelve patients with distal humerus fractures who underwent osteosynthesis using 3D preoperative planning were evaluated. Reduction shapes were evaluated by the angle between the diaphysis axis and a line connecting the vertices of the medial epicondyle and the lateral epicondyle (epicondyle angle), and the angle between the diaphysis axis and the articular surface (joint angle) in the coronal plane, and the distance between the anterior diaphysis and the anterior articular surface in the sagittal plane (anterior distance) based on 3D images of the distal humerus. In addition, the implant positions were evaluated by the positions of the proximal and posterior edge of the plate, and the angle of the plate to the epicondyle line. The reproducibility was evaluated by intra-class correlation coefficients of the parameters between pre- and postoperative images.

**Results:**

The intra-class correlation coefficients were 0.545, 0.802, and 0.372 for the epicondyle angle, joint angle, and anterior distance, respectively. The differences in the measurements between the preoperative plan and postoperative reduction were 2.1 ± 2.1 degrees, 2.3 ± 1.8 degrees, and 2.8 ± 2.0 mm, for the epicondyle angle, joint angle, and anterior distance, respectively. The intra-class correlation coefficients were 0.983, 0.661, and 0.653 for the proximal and posterior plate positions, and the angle to the epicondyle, respectively. The differences in the measurements between the preoperative plan and postoperative reduction were 3.3 ± 2.1 mm, 2.7 ± 1.7 mm and 9.7 ± 9.8 degrees, for the plate positions of proximal and posterior edge, and the angle of the plate to the epicondyle line, respectively. There were significant correlations for the epicondyle angle, joint angle, and plate positions.

**Conclusions:**

3D preoperative planning for osteosynthesis of distal humerus fracture was reproducible for the reduction shape of the coronal view and the plate positions. It may be helpful for acquiring practical images of osteosynthesis in distal humerus fractures.

**Level of evidence:**

Level III, a case–control study.

## Background

In surgical treatment of distal humerus fractures, locking plates are frequently used [1–3]. An advantage of locking plates is angle stability between the plate and screws [4–6]. Because of this angle stability, locking plates can be used in patients with highly comminuted fractures, unstable metadiaphyseal segments, and osteoporotic fractures [7]. Some reports suggested the importance of placing the plate in the optimum position to minimize the correction loss [8–10]. On the other hand, since the screw directions are fixed and distributed three-dimensionally, it is not easy to determine the optimum plate position and size or the length and directions of the screws during the surgery.

In the field of orthopedic surgery, it is becoming more common to introduce computer-aided technology [11–17]. Regarding 3D preoperative planning for fracture management, there have been some attempts to prepare a preoperative plan for osteosynthesis by creating 3D models or 3D images of fracture parts [16–21]. In complicated fracture patterns, such as pelvic fractures, 3D models were useful for determining surgical approaches and reduction techniques and appropriate internal fixation [16]. The clinical outcomes after using virtual surgical simulation and 3D printing for proximal humerus fractures were better than those in a conventional treatment [18]. Another report suggested that 3D preoperative plans for plate positions were reproducible in the osteosynthesis of proximal humerus fractures [19]. It was also found that a 3D preoperative plan was useful for optimizing screw selections in the osteosynthesis of distal radius fractures [20].

**Fig. 1:**
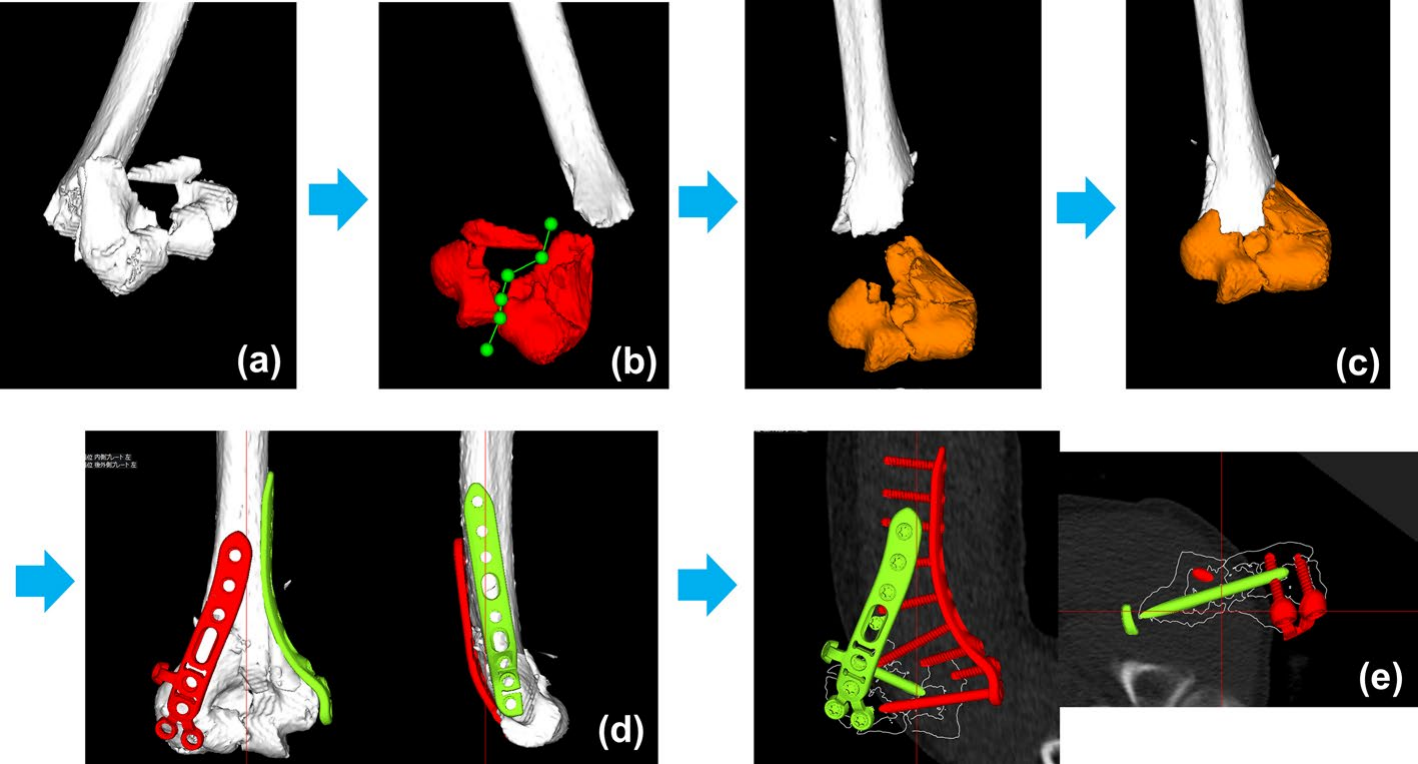
Example image of fracture reduction and implant placements. **a** After importing the DICOM images to the software, a 3D image of the distal humerus was made. **b** Fracture fragments were segmented according to the fracture lines. **c** Each fragment was repositioned in accordance with fracture lines. **d** The plate sizes and placements were simulated. **e** The screw sizes were determined and checked with axial, coronal, and sagittal views of the repositioned bone outline

Distal part of humerus has a complex bone shape with irregularities, and it is difficult to predict the implants’ position suitable for the fracture type. Because of olecranon and coronoid fossa, the trochlea of distal humerus has a very narrow space to insert locking screws. The screws must be carefully directed through this trochlea. In addition, if combinations of plates are selected (i.e., medial and lateral, or medial and posterolateral plates), the screws from the plates in different directions may interfere and lead to poor fixation of distal fragments. To reproduce anatomical reduction and appropriate implant placement/choices during osteosynthesis for elbow fractures, we developed a 3D preoperative planning system (Fig. 1). This system allows the physician to visualize the three-dimensional shape of the fracture, simulate reduction, place the implants, and check the appropriate implant choices in virtual space before surgery. In this study, we performed a trial simulation of reduction and fixation from CT images of previous cases who underwent osteosynthesis on distal humerus fractures, and verified the required functions for the 3D preoperative planning system. In addition, we evaluated the clinical application and reproducibility of the 3D preoperative planning in osteosynthesis for distal humerus fractures. The objective of this study was to assess the usability and reproducibility of 3D preoperative planning for the osteosynthesis of distal humerus fractures by comparing pre- and postoperative reduction and implant choices.

## Results

**Fig. 2:**
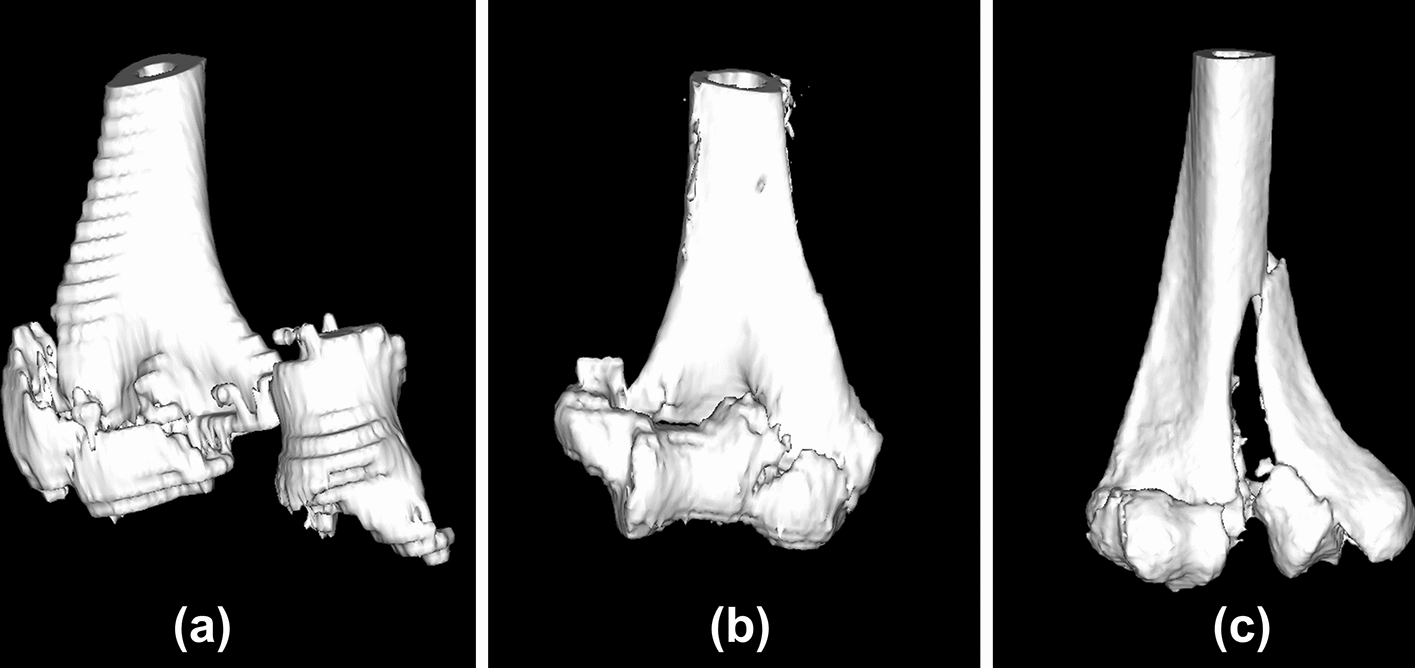
Comparison of 3D images created from CT images with different slice widths. **a** 3-mm slice width, **b** 2-mm slice width, **c** 1-mm slice width

In the trial phase, 15 distal humerus fracture cases were evaluated. There were four cases of CT images with 1-mm slice width, seven cases with 2-mm slice width, and four cases with 3-mm slice width. Strong adhesion present at the fracture-fragment interfaces led to failure of automatic partitioning in the 3-mm slice width cases. In these cases, manual etching of the fracture apertures was needed. For CT slices of more than 2-mm thickness, it was difficult to separate the fragments according to the exact fracture lines (Fig. 2). A grouping function was added because sometimes bone fragments were separated at different locations from the fracture lines. It was found imaging slices of less than 2 mm were favorable to simulate reduction for the fragments.

**Fig. 3:**
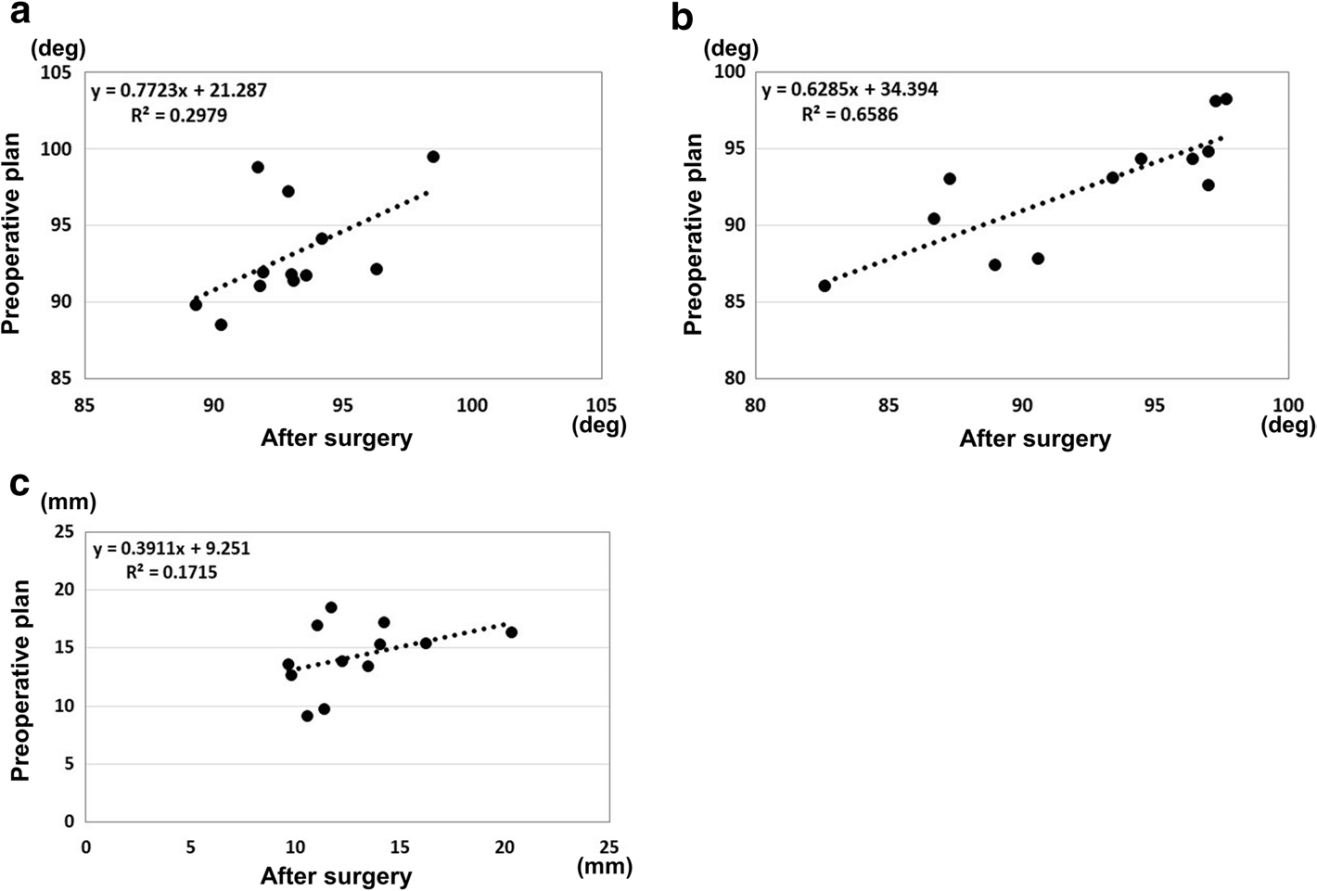
Reduction accuracy results. **a** Epicondyle angle, **b** Joint angle, **c** Anterior distance

**Fig. 4:**
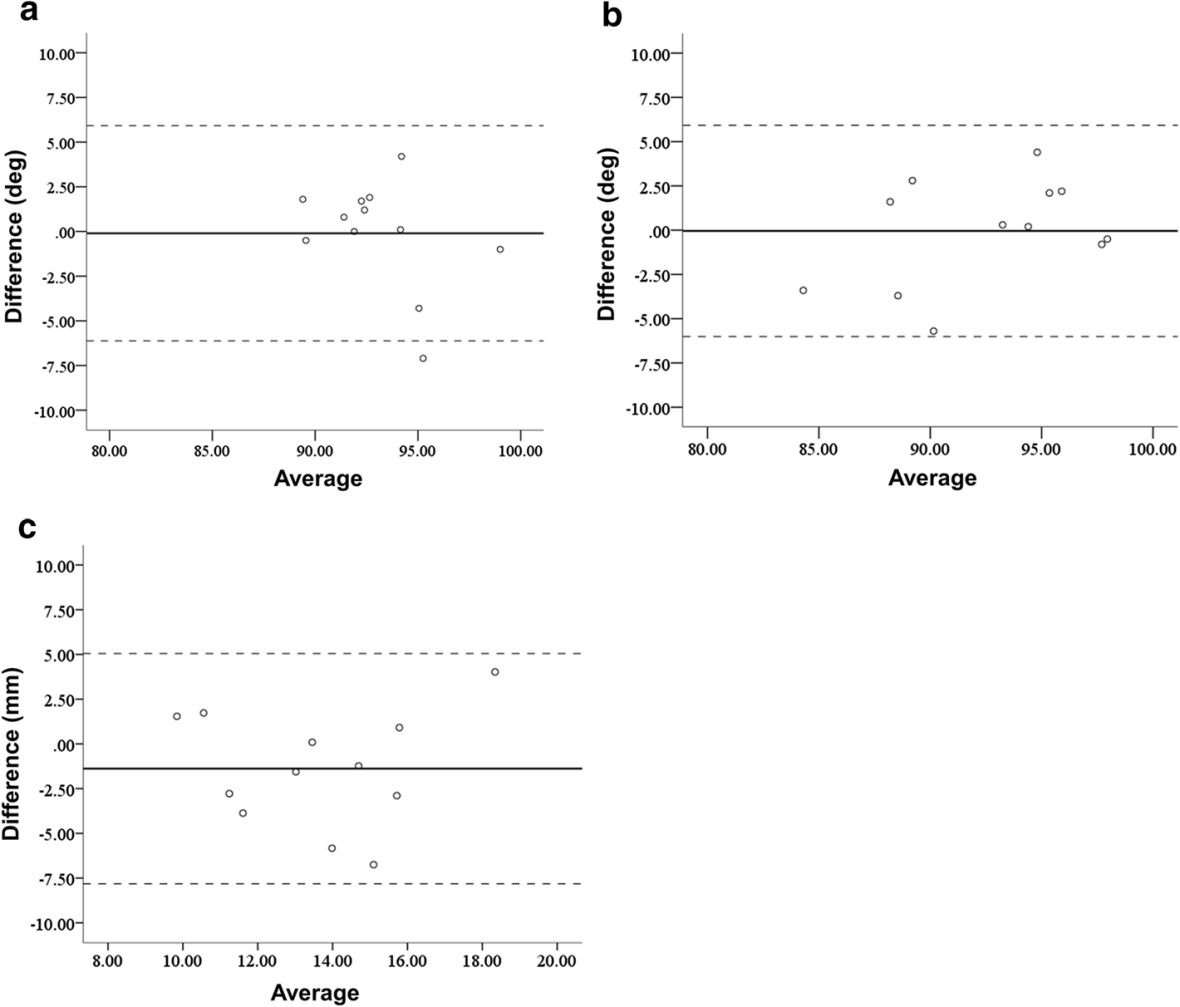
Results of Bland–Altman plot for the reduction parameters. The solid line represents the mean of the difference in the two measurements. The limits of agreement are shown by a dotted line from − 2 SD to +2 SD. **a** Epicondyle angle, **b** Joint angle, **c** Anterior distance

In the clinical application phase, there were three patients with A1, two with A2, one with A3, two with C1, one with C2, and three with C3 fractures in the AO classification. The average operation time was 200 min (range 112–274 min). The average fluoroscopy time was 6.3 min per patient (range 2.2–10.8 min). Mean blood loss was 123.6 mL (less than 10 mL up to 630 mL). We applied double plates in nine cases and a single plate in three cases. The planned sizes of plates were used for all cases. For double plates, a combination of medial–lateral plates was used in seven cases, and a combination of medial–posterolateral plates was used in two cases. There were no adverse events during the treatment process. The results for the reduction accuracy parameters are shown in Figs. 3 and 4. The ICC for the epicondyle angle was 0.545 (*P* = 0.02, moderate). The ICC for the joint angle was 0.802 (*P* < 0.01, excellent). The ICC for the anterior distance was 0.372 (*P* = 0.09, poor). The differences in the measurements between the preoperative plan and postoperative reduction were 2.1 ± 2.1 degrees, 2.3 ± 1.8 degrees, and 2.8 ± 2.0 mm, for the epicondyle angle, joint angle, and anterior distance, respectively. Bland–Altman plots demonstrated good reproducibility for all reduction parameters.

**Fig. 5:**
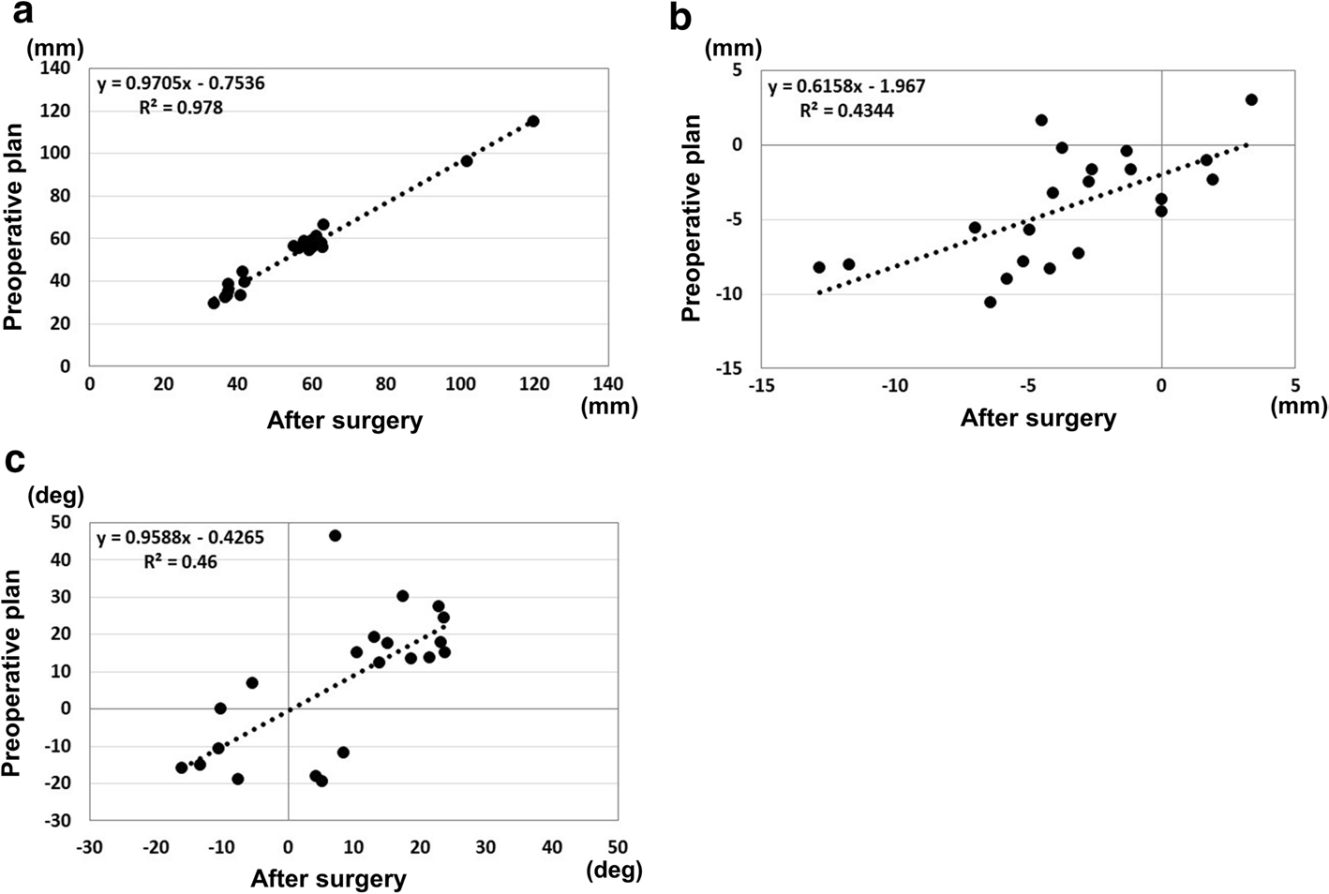
Implant placement results. **a** Results for the distance between the proximal edge of the plate and the proximal edge of the olecranon fossa. **b** Results for the distance between the posterior edge of the plate and the posterior edge of the bone. **c** Results for the angle between the lines of the plate surface and the epicondyle line

**Fig. 6:**
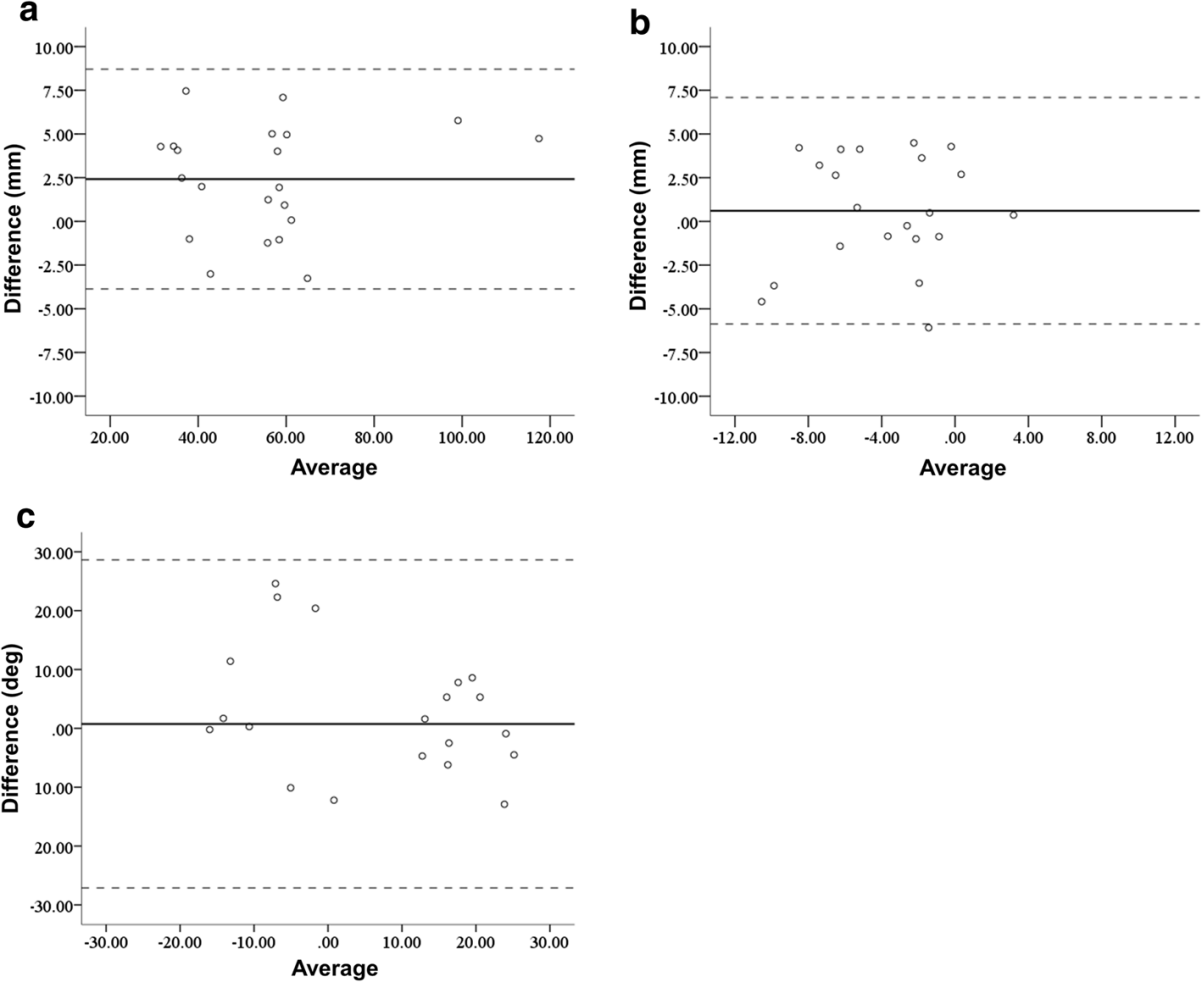
Results of Bland–Altman plot for the implant placement parameters. The solid line represents the mean of the difference in the two measurements. The limits of agreement are shown by a dotted line from − 2 SD to +2 SD. **a** Distance between the proximal edge of the plate and the proximal edge of the olecranon fossa. **b** Distance between the posterior edge of the plate and the posterior edge of the bone. **c** Angle between the lines of the plate surface and the epicondyle line

The results for the plate placement parameters are shown in Figs. 5 and 6. The ICC for the distance between the proximal edge of the plate and the proximal edge of the olecranon fossa was 0.983 (*P* < 0.01, excellent). The ICC for the distance between the posterior edge of the plate and the posterior edge of the bone was 0.661 (*P* < 0.01, good). The ICC for the angles between the plate surface and the epicondyle line was 0.653 (*P* < 0.01, good). The differences in the measurements between the preoperative plan and postoperative reduction were 3.3 ± 2.1 mm, 2.7 ± 1.7 mm and 9.7 ± 9.8 degrees, for the plate positions of proximal and posterior edge, and the angle of the plate to the epicondyle line, respectively. Bland–Altman plots demonstrated good reproducibility for all plate placement parameters.

## Discussion

In this study, we tried to develop a digital preoperative planning system for the osteosynthesis of distal humerus fractures. In the trial application phase, it was found less than 2-mm slice of CT image is necessary for the reduction simulation for distal humerus fractures. In general, a CT slice width of 5 mm is used in the evaluation of the thoracic and abdominal organs. On the other hand, in musculoskeletal CT scan, a slice width of 0.5 mm to 5 mm is selected depending on the site. For the preoperative simulation, it is necessary to identify where each bone fragment was originally located. Although the radiation exposure dose is smaller when imaging with a larger slice width, it was difficult to identify the fracture lines and fragments. Strong adhesion presented at the fracture-fragment interfaces when trying to perform separation and reduction simulation with the 3-mm slice imaging condition. Although there were no clear criteria regarding the slice width, it was shown that imaging with a 2-mm slice width or less was desirable for the preoperative planning of the distal humerus fractures.

With preoperative planning based on 2D images, it was difficult to understand rotational deformity. Another difficult task was in the transfer of the 2D preoperative plan to the actual surgery. These days, anatomical locking plates are widely used for distal humerus fractures. These plates are well designed and are considered to fit the average patient’s anatomy. However, they have been shown to lead to considerable positioning errors in individual patients. This may be because of the difficulty in predicting appropriate positions of implants three-dimensionally. The 3D preoperative planning system for distal humerus fractures used in this study can visualize the reduction process and check the implant sizes and placements. In the results of reduction shape at clinical application phase, the parameters in the coronal view (the epicondyle and joint angles) showed better reproducibility than the parameter in the lateral view (anterior distance). This may be because the reduction was performed mainly while looking at the anteroposterior image of the fluoroscopy during the surgery. Since the reconstructions of distal humerus anterior tilt are important to obtain better range of flexion for the elbow joint, the low reproducibility of the anterior distance needs to be improved. In the process of reduction simulation, it was possible to distinguish the fragments that could be fixed and the fragments that could not. Therefore, after the simulation, it was able to concentrate on the reduction of the bone fragments to be fixed with confidence during the surgery. In the results of plate positions, all parameters showed significant correlations. Especially, the distance between the proximal edge of the plate and the proximal edge of the olecranon fossa showed higher accuracy. This is because the surgery was performed while comparing the preoperative image with the fluoroscopic image for the distance from the articular surface of distal humerus to the distal end of the plate. The 3D preoperative plan gives a practical image of the plate positions. In addition, the surgeon’s images of reduction and implant placements can be shared with other medical staff involved in the surgery. These are practical benefits of 3D preoperative planning.

For fractures involving comminution of the articular surface at the distal part of the humerus, internal fixation with a double plate is often chosen. In such cases, the combination of a medial and lateral, or medial and posterolateral, double plate is common. To achieve sufficient primary stability, the number of screws inserted into the distal fragments is important [22–24]. At least two screws of sufficient length should be placed in the distal fracture fragments of the medial and lateral condyle. However, the screws from the medial and lateral, or medial and posterolateral condyle, sometimes overlap, and suitable length screws cannot be inserted. As a result, insufficient fixation of distal fragments may cause postoperative reduction loss [22, 25, 26]. The simulation was useful to evaluate appropriate plate positions and to avoid the overlap of screws. This process makes it possible to know in advance the plate position where the screws entering the distal part of the humerus do not interfere. In addition, by measuring the screw length before surgery, comparison and reconfirmation with intraoperative measurements can be done. This may be useful to prevent complications such as joint perforation by reducing choices of excessive screw length and deficiencies. In this regard, 3D preoperative planning may be useful to prevent postoperative correction loss by giving a firm fixation.

There were some limitations in this study. First, this was not a comparative study. The benefits of 3D preoperative planning should be evaluated by a comparative study of the clinical outcomes between 3D planning and conventional 2D planning. Second, this 3D preoperative planning cannot adapt to the in situ bending of the plates. Although this system can simulate screw angle variations, some extra functions may need to be added to make the system easier for clinical use. Third, there were no functional assessments in this study. There were several different methods, directly or indirectly informing about expected postoperative placement of implants and tissues or organ function [18, 27–29]. Future study will require functional assessment in comparison to the reduction shape and implant placement. In addition, the evaluations may need to be confirmed using other imaging techniques. Finally, it takes about 20 to 30 min to create the 3D image of the reduction and implant placement using the software. Shortening of this working time needs to be considered. Future tasks include simplifying the reduction of comminuted bone fragments at the joint surfaces, establishing a 3D verification method of reduction accuracy and reproducibility of implant positions, installing implant data from different makers, and reducing the CT scan radiation exposure dose. After solving these problems, it will be necessary to investigate the clinical significance of 3D preoperative planning for fractures around the elbow joint.

## Conclusions

3D preoperative planning system for the elbow joint fractures was developed. It was found less than 2-mm slice CT imaging is necessary for the fracture reduction simulation. For the reduction accuracy, the parameters in the coronal view (the epicondyle and joint angles) showed higher accuracy than the parameter in the lateral view (anterior distance). In addition, the reproducibility of plate positions showed significant correlations for all coronal, lateral, and axial view parameters. It suggests that 3D preoperative planning for osteosynthesis of distal humerus fracture is reproducible for the reduction shape and plate positions. 3D preoperative planning of elbow fracture is considered to have advantages of simulating fracture reduction, and giving a practical image of the implants placement and choices in a virtual space.

## Methods

This study protocol was approved by our Institutional Review Board (No. 14-21). This was a case–control study (level of evidence: level III). This study was registered as NCT04349319 at ClinicalTrials.gov (registered 15 April, 2020—retrospectively registered). Written informed consent was obtained from all study participants.

### Trial application phase

From our database of previous distal humerus fracture patients who were treated with locking plates between June 2011 and October 2017, we investigated which CT scan slice width was appropriate for 3D preoperative planning. We retrospectively extracted elbow joint CT images with different slice widths of 1 mm, 2 mm, and 3 mm. The preoperative plans were simulated using software newly developed by the authors (Zed-Trauma, LEXI Co., Ltd. Tokyo, Japan). The planning is based on digital imaging and communications in medicine (DICOM) data from CT scans. After importing the DICOM datasets to the software, 3D images of the fracture site from different CT data slice widths were created. Each distal humerus fracture was segmented according to the main fracture fragments using the cut function. Each fragment was repositioned in accordance with fracture lines. After repositioning the fragments, the bone shape was checked three-dimensionally. Implant choice and placement were also simulated with these images. The capacity for image characterization on 3D preoperative planning software and possibility of simulation were evaluated with image data from different slice widths.

### Clinical application phase

Twelve patients with distal humerus fractures who underwent osteosynthesis using 3D preoperative planning (seven females, five males, mean age 66.0 years, age range 35–87) were evaluated. Patients were excluded if they reported a previous history of traumatic injuries to the elbow. The patients were treated between October 2017 and May 2019. According to the preoperative CT scans, fractures were classified using the AO classification system. Preoperative planning was performed to determine the reduction, placement and choice of implants. DICOM datasets from the CT scans were imported into the software and 3D models of the distal humerus were created. Each distal humerus fracture was segmented according to the main fracture fragments using the cut function. Each fragment was repositioned in accordance with fracture lines. After repositioning the fragments, the bone shape was checked three-dimensionally. A 3D template of the implants was used to simulate placing plates/screws of different sizes. The A.L.P.S. elbow plating system (Zimmer Biomet Holdings, Inc., Warsaw, USA) was used in this study. This plate system has medial, lateral, and posterolateral plates for the distal humerus, and 64–210 mm (7–21 holes) for the plate length. Screw lengths from 10 to 60 mm (3.5-mm diameter) are available. Computer-aided design models of different-size implants were installed in the software. The criteria for plate selection were as follows: (1) the proximal part of the plate crosses the fracture line and reaches the trunk of the humerus disphysis, and the screw can be inserted with a minimum of three holes in the proximal part of the humerus, (2) the screws can fix the main bone fragments at the distal part of the humerus and reconstruct the joint surface, (3) the insertion direction of the distal screws does not result in penetration of the joint surface.

**Fig. 7:**
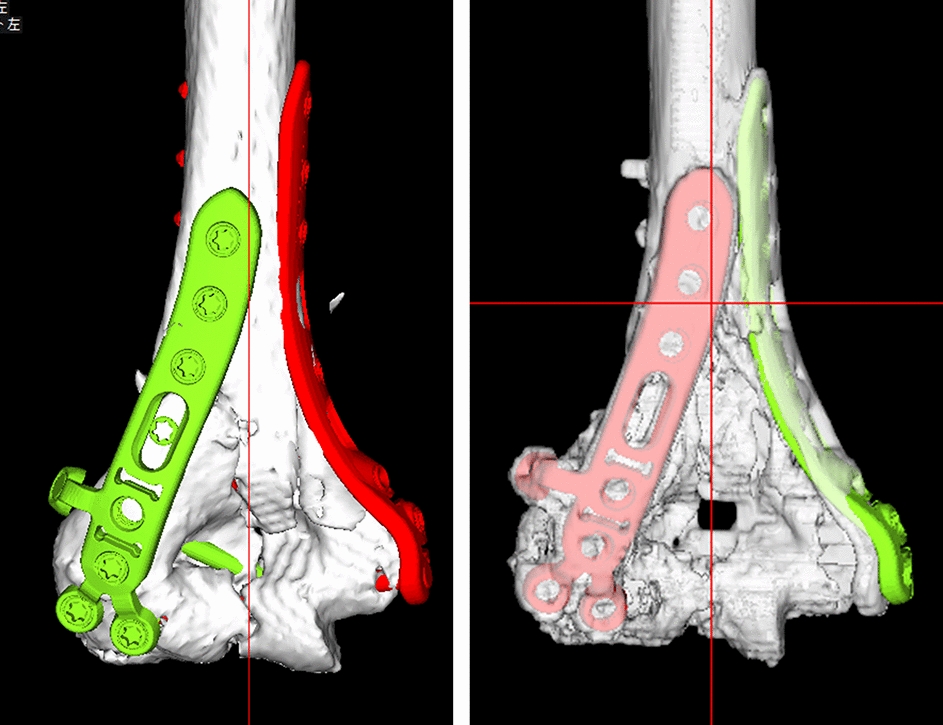
Example preoperative plan and post-surgery images. **a** Preoperative plan. **b** After surgery

After the preoperative planning, osteosynthesis was performed under general anesthesia. The surgeons tried to reproduce the repositions by comparing the planned 3D image with the fluoroscopic image. The positions of implants were checked by the distances from the margin of the implant to the margin of the humerus, and compared between the planned 3D image and the fluoroscopic image. The screw sizes were finally determined by intraoperative measurements in reference to the preoperative plan. The surgeries were performed by several orthopedic surgeons and one hand surgery specialist. After surgery, we did CT scans and verified the accuracy of the reduction, implants’ choices and placements (Fig. 7).

### Evaluations

**Fig. 8:**
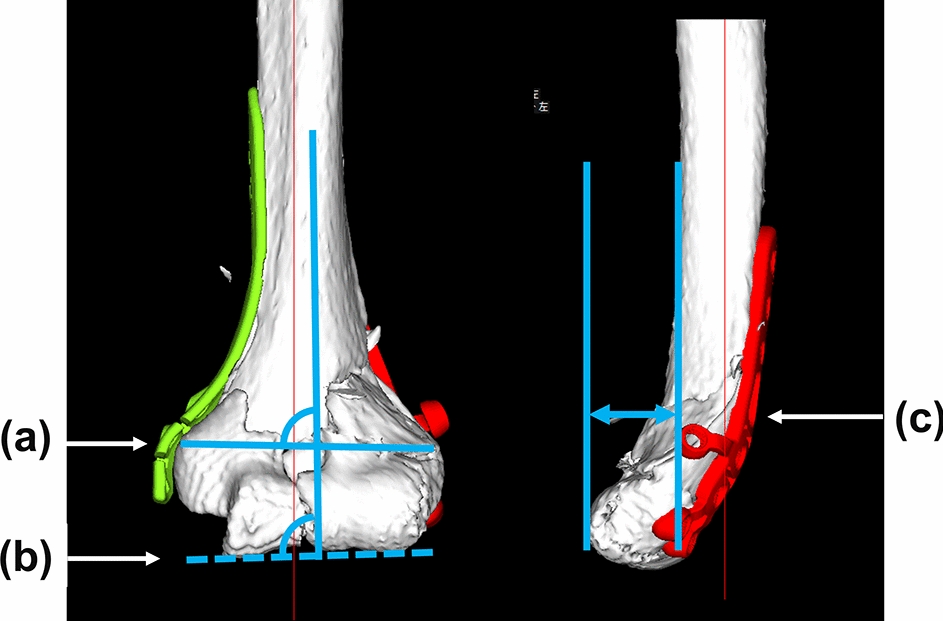
Evaluation of reduction accuracy. **a** Angle between the diaphysis axis and a line connecting the vertices of the medial epicondyle and the lateral epicondyle (epicondyle angle). **b** Angle between the diaphysis axis and the articular surface (joint angle). **c** Distance between the anterior diaphysis and the anterior articular surface in the sagittal plane (anterior distance)

**Fig. 9:**
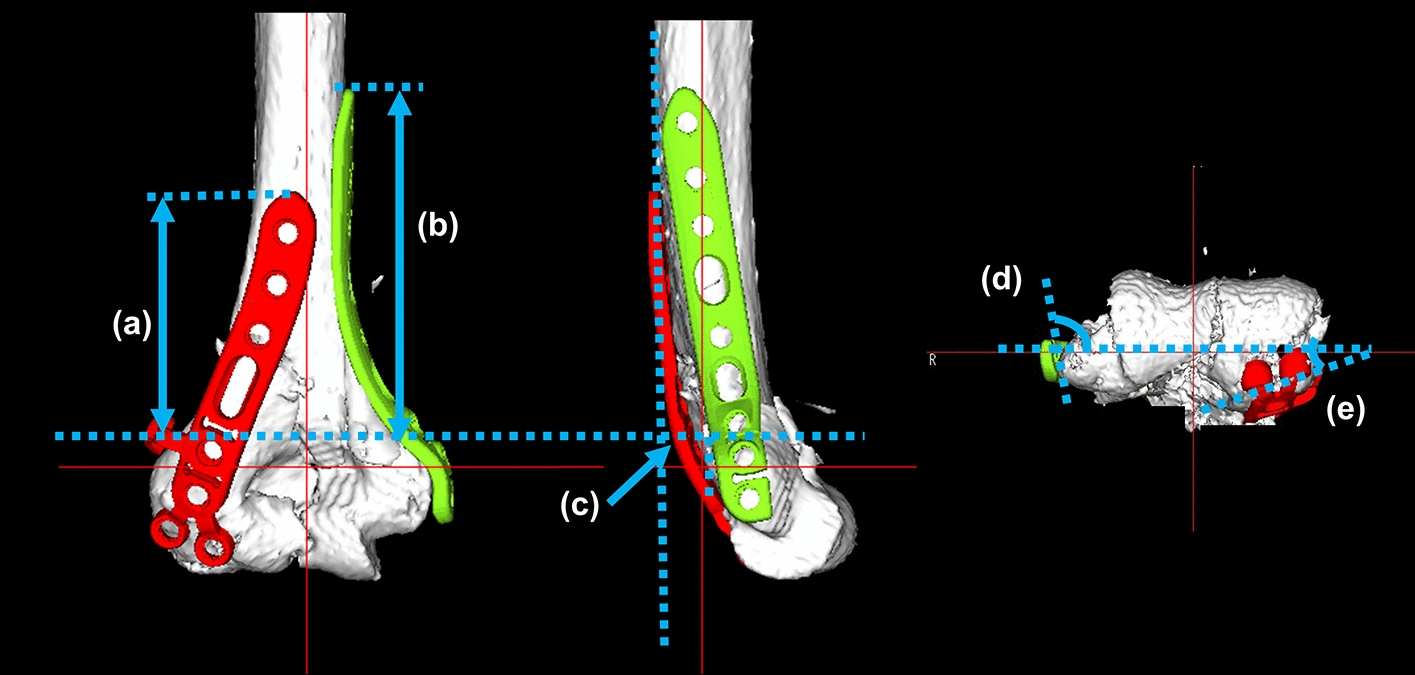
Evaluation of implant placements. **a**, **b** Distance between the proximal edge of the plate and the proximal edge of the olecranon fossa. **c** Distance between the posterior edge of the plate and the posterior edge of the bone at the olecranon fossa level. **d**, **e** Angles between the lines of the plate surface and the line connecting the vertices of the lateral and medical epicondyles

Operation time, blood loss, and fluoroscopy time were recorded intraoperatively. To evaluate the reproducibility of the 3D planning, preoperative planning and postoperative reductions were compared by measuring 3D image parameters (Figs. 8 and 9). The reductions were evaluated by the angle between the diaphysis axis and a line connecting the vertices of the medial epicondyle and the lateral epicondyle (Fig. 8a, epicondyle angle) and the angle between the diaphysis axis and the articular surface (Fig. 8b, joint angle) in the coronal plane and the distance between the anterior diaphysis and the anterior articular surface in the sagittal plane (Fig. 8c, anterior distance) for the distal humerus 3D images. In addition, the reproducibility of plate positions was evaluated with coronal, sagittal, and axial 3D image views. In the coronal view, the distance between the proximal edge of the plate and the proximal edge of the olecranon fossa was measured for both the medial and lateral plates (Fig. 9a, b). In the sagittal view, the distance between the posterior edge of the plate and the posterior edge of the bone at the olecranon fossa was measured (Fig. 9c). In addition to the plate positions, the angles between the lines of the plate surface and the line connecting the vertices of the lateral and medical epicondyles were measured in the axial view (Fig. 9d, e).

### Statistical analysis

For the reproducibility of the reduction and the plate placements, intra-class correlation coefficients (ICC) for the parameters between the preoperative plan and the postoperative reductions were evaluated. The results are expressed as the mean ± standard deviation. To assess the accuracy of the implant placements, the ICCs for the plate placements between the preoperative plan and the postoperative images were evaluated. According to a previous recommendation [30], ICC less than 0.40 was considered poor reproducibility, between 0.40 and 0.59 was considered moderate, between 0.60 and 0.74 was considered good, and between 0.75 and 1.00 was considered excellent. *P* values of < 0.05 were considered significant.

In addition, Bland–Altman analysis was performed to evaluate differences between preoperative and postoperative measurements of the reduction and the plate placement. Bland–Altman plots are the plots of difference (the vertical axis demonstrates the difference between preoperative and postoperative measurements) and their average (the horizontal axis demonstrates the average for the preoperative and postoperative measurements). When most of the data points were situated between the limits of reproducibility represented by a dotted upper line (mean + 2 SD: standard deviation) and lower line (mean − 2 SD), this indicated good reproducibility. All statistical analyses were performed using BellCurve for Excel version 2.12 (SSRI Co., Tokyo, Japan) and IBM SPSS Statistics version 24 (IBM, Tokyo, Japan).

## Data Availability

The datasets analyzed during the current study are available from the corresponding author on reasonable request.

## Abbreviations

3D: Three-dimensional
2D: Two-dimensional
CT: Computed tomography
DICOM: Digital imaging and communications in medicine
ICC: Intra-class correlation coefficients
